# Identifying priority landscapes for conservation of snow leopards in Pakistan

**DOI:** 10.1371/journal.pone.0228832

**Published:** 2020-11-05

**Authors:** Shoaib Hameed, Jaffar ud Din, Hussain Ali, Muhammad Kabir, Muhammad Younas, Ejaz ur Rehman, Fathul Bari, Wang Hao, Richard Bischof, Muhammad Ali Nawaz

**Affiliations:** 1 Department of Zoology, Quaid-i-Azam University, Islamabad, Pakistan; 2 Snow Leopard Trust, Pakistan Program, Islamabad, Pakistan; 3 Institute of Biological Sciences, Faculty of Science, University of Malaya, Kuala Lumpur, Malaysia; 4 Department of Forestry and Wildlife Management, University of Haripur, Haripur, Pakistan; 5 School of Life Sciences, Peking University, Beijing, China; 6 Faculty of Environmental Sciences and Natural Resource Management, Norwegian University of Life Sciences, Ås, Norway; 7 Department of Biological and Environmental Sciences, Qatar University, Doha, Qatar; U.S. Geological Survey, UNITED STATES

## Abstract

Pakistan’s total estimated snow leopard habitat is about 80,000 km^2^ of which about half is considered prime habitat. However, this preliminary demarcation was not always in close agreement with the actual distribution—the discrepancy may be huge at the local and regional level. Recent technological developments like camera trapping and molecular genetics allow for collecting reliable presence records that could be used to construct realistic species distribution based on empirical data and advanced mathematical approaches like MaxEnt. The current study followed this approach to construct an accurate distribution of the species in Pakistan. Moreover, movement corridors, among different landscapes, were also identified through circuit theory. The probability of habitat suitability, generated from 98 presence points and 11 environmental variables, scored the snow leopard’s assumed range in Pakistan, from 0 to 0.97. A large portion of the known range represented low-quality habitat, including areas in lower Chitral, Swat, Astore, and Kashmir. Conversely, Khunjerab, Misgar, Chapursan, Qurumber, Broghil, and Central Karakoram represented high-quality habitats. Variables with higher contributions in the MaxEnt model were precipitation during the driest month (34%), annual mean temperature (19.5%), mean diurnal range of temperature (9.8%), annual precipitation (9.4%), and river density (9.2). The model was validated through receiver operating characteristic (ROC) plots and defined thresholds. The average test AUC in Maxent for the replicate runs was 0.933 while the value of AUC by ROC curve calculated at 0.15 threshold was 1.00. These validation tests suggested a good model fit and strong predictive power. The connectivity analysis revealed that the population in the Hindukush landscape appears to be more connected with the population in Afghanistan as compared to other populations in Pakistan. Similarly, the Pamir-Karakoram population is better connected with China and Tajikistan, while the Himalayan population was connected with the population in India. Based on our findings we propose three model landscapes to be considered under the Global Snow Leopard Ecosystem Protection Program (GSLEP) agenda as regional priority areas, to safeguard the future of the snow leopard in Pakistan and the region. These landscapes fall within mountain ranges of the Himalaya, Hindu Kush and Karakoram-Pamir, respectively. We also identified gaps in the existing protected areas network and suggest new protected areas in Chitral and Gilgit-Baltistan to protect critical habitats of snow leopard in Pakistan.

## Introduction

The snow leopard, *Panthera uncia* has obtained an iconic status worldwide and is treated as a flagship species of the Greater Himalayan ecosystem [1]. The species is native to the mountain ranges of Central and Southern Asia—some of the world’s most rugged landscapes [2–4]. It occurs in the Hindu Kush, Karakoram, Altai, Sayan, Tien Shan, Kunlun, Pamir, and outer Himalayan ranges, and smaller isolated mountains in the Gobi region [5–7]. Estimates of global range size vary from 1.2 million to over 3 million km^2^ [4] and the species is highly threatened throughout its range. A recent study estimated its occupied range to be about 2.8 million km^2^ [8], across 12 countries—Afghanistan, Bhutan, China, India, Kazakhstan, Kyrgyzstan, Mongolia, Nepal, Pakistan, Russia, Tajikistan, and Uzbekistan [9–11]. The potential range of snow leopard may also extend to northern Myanmar, but recent snow leopard presence in this area has not been confirmed [4].

Pakistan’s total estimated snow leopard habitat according to Roberts’ [12] range maps is approximately 80,000 km^2^, and about half of it is considered prime habitat [13]. However, this preliminary evaluation of the snow leopard’s distribution is based on expert judgments, anecdotal information, and topography. Consequently, these coarse distribution maps are not always in close agreement with the actual distribution—the discrepancy may be huge at the regional and global levels [9]. Accurate modelling of the geographic distribution of species is crucial to various applications in ecology and conservation [14–16]. Conservationists often need precise assessments of species’ ranges and current species distribution patterns. Furthermore, the range description is essential, but concrete identification of factors that restrict distributions is also critical to inform conservation management [17].

It is important to be aware of the variables constraining or facilitating species’ occurrence to avoid under prediction or over prediction of its distribution or habitat suitability [18] and much of these data are readily available, for example in the form of digital elevation models (https://www.usgs.gov) and global databases of climate (https://www.worldclim.org), productivity, and human impacts/infrastructure (http://www.fao.org). Ecological niche models (ENMs) and species distribution models (SDMs) are increasingly being used to map potential distributions of many species [15, 19, 20]. These models incorporate species occurrence data with climatic and other environmental variables to produce distribution maps of species [21]. During this process, the models also estimate species-specific environmental suitability across a given spatial extent [14]. Information about species distribution and habitat suitability can in turn be used to design scientific surveys and plan conservation interventions [22].

Many models like BIOCLIM, BLOMAPPER, DIVA, DOMAIN, CLIMEX, GAM, GLM, and GARP have been used in species distribution modelling [23–27], but Maximum Entropy (MaxEnt) is widely used in habitat suitability modeling due to its accuracy, additional descriptive properties [28], and better predictive functions [29]. MaxEnt estimates the probability of the presence of a species based on occurrence records and randomly generates background points by finding the maximum entropy distribution [22, 30]. These models can use either presence/absence data or presence-only data. The use of presence/absence data in wildlife management and biological surveys is widespread [31]. However, species absence data are often unavailable or believed to be too difficult to interpret [32]. Nevertheless, SDMs trained on presence-only data are frequently used in ecological research and conservation planning [20, 28]. Presence-based modelling methods only require a set of known occurrences together with predictor variables such as topographic, climatic, and biogeographic variables [33].

Connectivity among habitats and populations is another critical factor that influences a variety of ecological phenomena, including gene flow, metapopulation dynamics, demographic rescue, seed dispersal, infectious disease spread, range expansion, exotic invasion, population persistence, and maintenance of biodiversity [34–40]. Preserving and restoring connectivity is one of the top conservation priorities and conservation organizations are devoting substantial resources to accomplish these goals [41, 42]. A reliable, efficient, and process-based approached is required to achieve this objective in complex landscapes. A new class of ecological connectivity models based on electrical circuit theory was introduced by McRae et al. [43]. Resistance, current, and voltage calculated across graphs or raster grids can be associated with ecological processes like; individual movement and gene flow, that take place across large population networks or landscapes.

**Fig 1:**
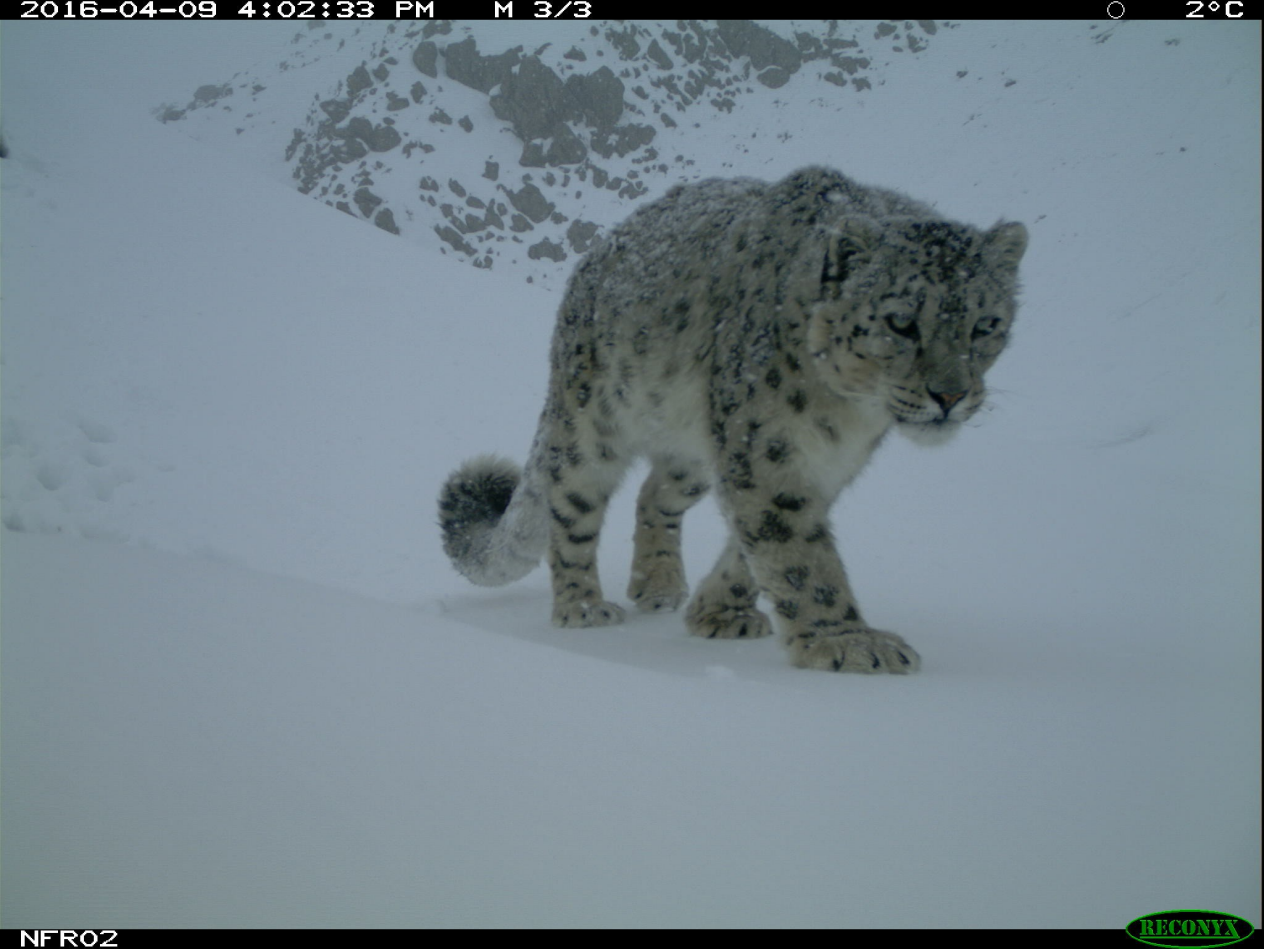
Photo of an adult snow leopard taken with a camera trap in Hopper-Hisper Valley in northern Pakistan during non-invasive surveys in 2016.

Given the multitude of threats to snow leopards and their habitat, it is imperative that comprehensive landscape-level conservation strategies be developed that are based on reliable information on species survival requirements. A global strategy to safeguard snow leopards and the vast ecosystem they inhabit—which includes 12 nations and supports 1 billion people—has already been established: The Global Snow Leopard Ecosystem Protection Program (GSLEP). Its overall aim is to secure at least 20 snow leopard model landscapes across the species’ range by 2020 [44]. Under the GSLEP initiative, the selection of model landscapes requires a clear understanding of areas that represent the species’ prime habitat so that conservation efforts in the next decade can focus on securing areas that hold or have the potential to hold larger populations; at least 100 breeding age snow leopards. Recent technological developments like camera trapping (Fig 1) and molecular genetics allow for the collection of reliable presence records across large spatial expanses that could be used to construct realistic species distribution maps. Relying on these technologies, this study aimed to support the GSLEP initiative by identifying core habitats and movement corridors in Pakistan.

## Materials and methods

### Study area

**Fig 2:**
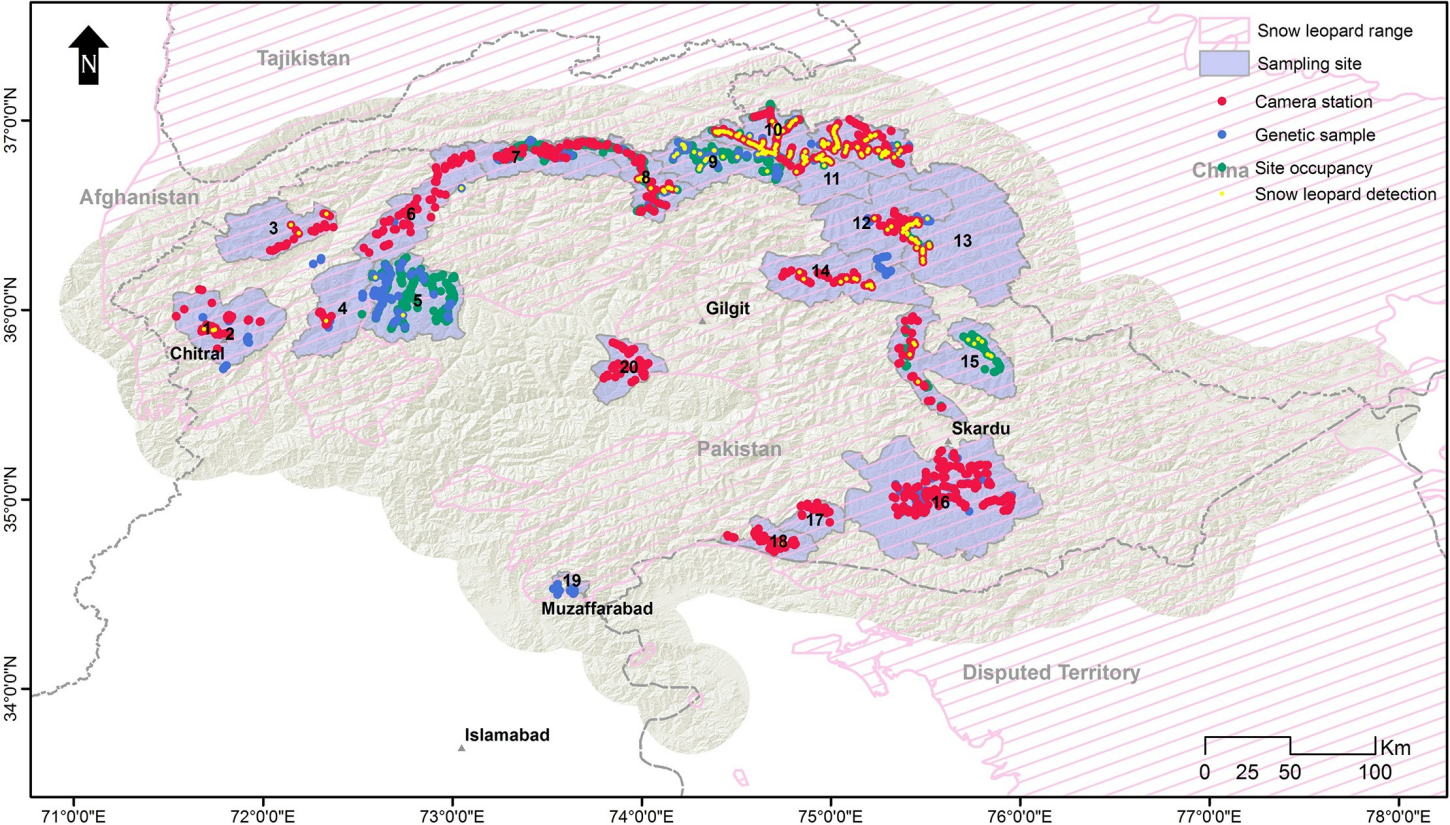
Map of study area showing sampling sites and IUCN range of snow leopard in Pakistan. 1 = Chitral Gol National Park, 2 = Chitral and Surrounding-Tooshi, 3 = Terich, 4 = Laspur, 5 = Phandar, 6 = Yarkhun, 7 = Broghil National Park, 8 = Qurumber National Park, 9 = Chapursan, 10 = Misgar, 11 = KVO-Sukhtarabad, 12 = Shimshal, 13 = Khunjerab National Park, 14 = Hopper-Hisper, 15 = Basha-Arandu, 16, Deosai National Park and surroundings, 17 = Kalapani-Astore, 18 = Musk Deer National Park, 19 = Machiara National Park, 20 = Khanbari.

The study focused on the known snow leopard range in Pakistan [12, 13] which encompasses four high mountain ranges; the Himalaya, Karakoram, Pamir, and the Hindu Kush spread across three administrative units, i.e. Khyber Pakhtunkhwa (KP), Gilgit-Baltistan (GB), and Azad Jammu and Kashmir (AJK). Targeting major protected areas and other potentially suitable habitats, we surveyed 20 sites with a collective area of around 31,000 km^2^ (Fig 2). The surveyed areas constitute 39% of reported snow leopard habitat in Pakistan (80,000 km^2^) (Fig 2) [45].

Their high altitudes and sub-zero temperatures make our study area one of the most heavily glaciated parts of the world outside the Polar Regions. The Western Himalayan Range is situated in AJK and GB to the south and east of the Indus River. The Hindukush rise southwest of the Pamirs. The Karakoram Range covers the borders between three countries in the regions of GB in Pakistan, Ladakh in India, and the Xinjiang region in China. They are considered to extend from the Wakhjir Pass at the junctions of the Pamirs and Karakoram to the Khawak Pass north of Kabul. The mountains of Pakistan are relatively densely populated despite harsh geographic and climatic conditions. Nevertheless, the special ecological conditions and remoteness of these mountainous areas also support unique biodiversity of plants and animals. Climatic conditions vary widely across the study area, ranging from the monsoon-influenced moist temperate zone in the western Himalayas to the semi-arid cold deserts of the northern Karakorum and Hindu Kush. Four vegetation zones can be differentiated along with the altitudinal ascents: alpine dry steppes, subalpine scrub zones, alpine meadows, and permanent snowfields. Various rare and endangered animals occur in the study area, including the snow leopard (*Panthera uncia*), grey wolf (*Canis lupus*), brown bear (*Ursus arctos*), Asiatic black bear (*Ursus thibetanus*), Himalayan lynx (*Lynx lynx*), Himalayan Ibex (*Capra ibex sibirica*), blue sheep (*Pseudois nayaur*), flare-horned markhor (*C*. *f*. *cashmirensis*), musk deer (*Moschus chrysogaster*), Marco Polo sheep (*Ovis ammon polii*), Ladakh urial (*Ovis orientalis vignei*) Pallas’s cat (*Otocolobus manual*) and woolly flying squirrel (*Eupetaurus cinereus*).

### Data collection

Presence records were collected using three methods: camera trapping, sign surveys and genetic sampling. Camera trapping is being increasingly adopted for the monitoring of shy and rare wildlife [46–48]. We deployed 806 camera stations in Chitral Gol National Park (CGNP), the buffer areas of CGNP and Tooshi Game Reserve (TGR), TGR, Laspur Valley, Khunjerab National Park (KNP), Shimshal, Khunjerab Villagers Organization (KVO) area, Qurumber National Park, Broghil National Park, Deosai National Park, Yarkhun Valley, Misgar, Kalapani-Astore, Musk Deer National Park, Khanbari Valley, Terich Valley, Hopper-Hisper, Basha-Arandu and buffer areas of Central Karakoram National Park (CKNP), during the period 2006–2017 (Fig 2). These cameras remained active for more than 22,000 trap-days in the field with an average of 28.7 days per camera and a standard deviation of 15.6. The camera brands used were CamTrakker™ (Ranger, Watkinsville, GA, USA) and Reconyx^TM^ (HC500 Hyperfire^TM^ and PC900 Hyperfire^TM^; Reconyx, Holmen, Wisconsin, USA). The sites for camera installation were selected near tracks, scrapes, scats, and other signs. A minimum aerial distance of 1 km was kept between the two nearest camera stations. Installation and setup followed guidelines provided by Jackson et al. [47]. The majority of the camera stations were supplied with a different type of lures—castor, skunk, and fish oil—to enhance capture probability [49].

Site Occupancy based sign surveys were conducted in KNP-KVO-Shimshal, Qurumber-Broghil national parks, Misgar-Chapursan, Phandar Valley, and Basha-Arandu from 2010 to 2017. Each study area was divided into small grids cells of 5 × 5 km—except in KNP-KVO-Shimshal where we kept grid size to 10 × 10 km on GIS maps. Each grid cell (site) was approached by GPS and multiple points were led to search the signs for snow leopards. A total of 193 sites with 1,607 repeat survey points were searched for signs of snow leopards (Fig 2). The presence was detected through five types of signs (scrapes, pugmarks, faeces, scent spray, and claw marks). However, in this analysis, we only included scrapes and pugmarks to confirm snow leopard presence, as these are considered the most reliable [50].

Faecal samples were collected from 2009 to 2013 during the sign and camera trap surveys. We collected over 1,000 faecal samples of all carnivore species (Fig 2) encountered in the field and preserved them in 95% alcohol in 20 ml bottles. The DNA extraction was performed in a laboratory dedicated to the extraction of degraded DNA. Total DNA was extracted from c. 15 mg of faeces using the DNeasy Blood and Tissue Kit (QIAgen GmbH, Hilden, Germany) following the maker's guidelines with a small modification as explained by Shehzad et al. [51]. Blank extractions were performed to scrutinize contamination. Species identification was performed through next-generation sequencings (NGS) by amplifying DNA extract using primer pair 12SV5F (5’-TAGAACAGGCTCCTCTAG-3’) and 12SV5R (5’- TTAGATACCC CACTATGC-3’ targeting about 100-bp of the V5 loop of the mitochondrial 12S gene [48, 51] The sequence analysis and taxon assignation were done using OBITools as described in Shehzad et al. [51, 52].

Snow Leopard Foundation signed Memorandum of Understating (MoU) with the Provincial Wildlife departments which legally allowed for doing research in National Parks and other habitats.

### Data analysis

We used MaxEnt 3.3.3k [30] to predict snow leopard distribution in Pakistan. MaxEnt predicts species distribution using presence-only data and environmental variables and estimates species’ probability distribution by finding the probability distribution of maximum entropy, i.e. the most spread out or closest to uniform, subject to a set of constraints; mainly the possibility of over-fitting which limiting the capacity of the model to generalize well to independent data [30]. It is amongst the most popular species distribution modelling methods with more than 1,000 published usages since 2005 [15, 53]. MaxEnt has also surpassed other methods and exhibited higher predictive accuracy [23, 54].

We used a random seed option and kept 25% of data for random tests—25 replicates were run with typeset as a subsample. The rest of the settings were kept as default, which included a maximum of 10,000 randomly generated background points, 5,000 maximum iterations with a convergence threshold of 0.00001, and a regularization multiplier of 1.

#### Data preparation

We used snow leopard range with an added buffer of 30 km to model using MaxEnt. All environmental layers were converted to the same size (extent) and resolution, i.e. 1 × 1 km. Snow leopard occurrence points were also converted into a grid file. All environmental variables and presence points were then converted into ASCII files as required by MaxEnt, by using the ‘conversion’ tool in Arc GIS 10.2. Features in Maxent are derived from two types of environmental variables: continuous and categorical [33].

We considered 28 variables initially (Table 1), but removed highly correlated variables variables from the analysis, using Pearson correlation matrix [55]. After multicollinearity test, 11 environmental variables were retained (r < 0.70), including 4 bioclimatic variables (bio1, bio2, bio12, and bio14), distances from the river, roads and settlements, slope, ruggedness, soil, and a normalized difference vegetation index (NDVI) [48]. Bioclimatic variables were derived from the mean temperature, minimum temperature, maximum temperature, and precipitation to generate more biologically meaningful variables—these are often used in ecological niche modelling. Details of each variable used, and their sources are shown in Table 1. Records obtained via sign surveys, genetic sampling, and camera trapping were screened in SDMtoolbox, a tool of GIS, to remove spatially correlated data points, located within 5 km of each other, to guarantee independence [56–58]. After this selection, 98 unrelated locations were used in the analysis.

**Table 1:** List of environmental variables used in MaxEnt modelling.

*Environmental variable*	*Interpretation*	*Source*
bio1	Annual mean temperature	http://www.worldclim.org
bio2	Mean diurnal range (mean of monthly [max temp—min temp])	http://www.worldclim.org
bio3	Isothermality (Bio2/Bio7) (* 100)	http://www.worldclim.org
bio4	Temperature Seasonality (standard deviation *100)	http://www.worldclim.org
bio5	Max Temperature of Warmest Month	http://www.worldclim.org
bio6	Min Temperature of Coldest Month	http://www.worldclim.org
bio7	Temperature Annual Range (Bio5-Bio6)	http://www.worldclim.org
bio8	Mean Temperature of Wettest Quarter	http://www.worldclim.org
bio9	Mean Temperature of Driest Quarter	http://www.worldclim.org
bio10	Mean Temperature of Warmest Quarter	http://www.worldclim.org
bio11	Mean Temperature of Coldest Quarter	http://www.worldclim.org
bio12	Annual precipitation	http://www.worldclim.org
bio13	Precipitation of Wettest Month	http://www.worldclim.org
bio14	Precipitation of driest month	http://www.worldclim.org
bio15	Precipitation Seasonality (Coefficient of Variation)	http://www.worldclim.org
bio16	Precipitation of Wettest Quarter	http://www.worldclim.org
bio17	Precipitation of Driest Quarter	http://www.worldclim.org
bio18	Precipitation of Warmest Quarter	http://www.worldclim.org
bio19	Precipitation of Coldest Quarter	http://www.worldclim.org
alt	Elevation above sea level (m)	SRTM
slope	Slope of the area	derived from alt in Arc GIS 10.2
river	Density of rivers (m)	calculated in Arc GIS 10.2
road	Density of roads (m)	calculated in Arc GIS 10.2
settlement	Density of settlements (m)	calculated in Arc GIS 10.2
ndvi (MODIS)	Normalized difference vegetation index	NASA: http://modis-land.gsfc.nasa.gov/vi.html
soil	Digital soil map of the world	FAO, 2003
vrmint	Vector ruggedness measure	Generated from SRTM 90m DEM by the Center for Nature and Society, Peking University using the Terrain Ruggedness (VRM) Tool
glc2000	Global landcover 2000	USGS: http://edcsns17.cr.usgs.gov/glcc

#### Model evaluation

The fit or accuracy of the model should be tested, for every modelling approach, to determine its prediction. This can be done in two ways in MaxEnt: 1) through receiver operating characteristic (ROC) plots, and 2) through defined thresholds [18]. We used both approaches to determine model accuracy.

Model robustness is commonly evaluated by area under the curve (AUC) values of the ROC [59] that range from 0 to 1—AUC values in the range 0.5–0.7 are considered low, 0.7–0.9 moderate, and 0.9, high [60, 61]. Values close to 0.5 indicate a fit no better than that expected by random, while a value of 1.0 indicates a perfect fit. It is also possible to have values less than 0.5—this indicates that a model fits worse than random [62]. It is a graded approach for evaluating model fit that verifies the probability of a presence location being graded higher than random background locations that serve as pseudo-absences for all analyses in MaxEnt [30]. The AUC quantifies the significance of this curve and we used its values to determine model accuracy. ROC is a plot of the sensitivity vs. 1-specificity over the entire range of threshold values between 0 and 1 [63]. Using this method, the commission and omission errors are, therefore, weighted with equal importance for determining model performance [64].

Another approach entails selecting thresholds to determine sites that are considered suitable or unsuitable for the species of interest. These thresholds are established by maximizing sensitivity while minimizing specificity [30, 63]. The proportion of sites that are precisely categorized as suitable locations can be compared to the proportion of unsuitable sites to verify model accuracy. We checked our model output against different defined thresholds and selected the one with the lowest error.

**Fig 3:**
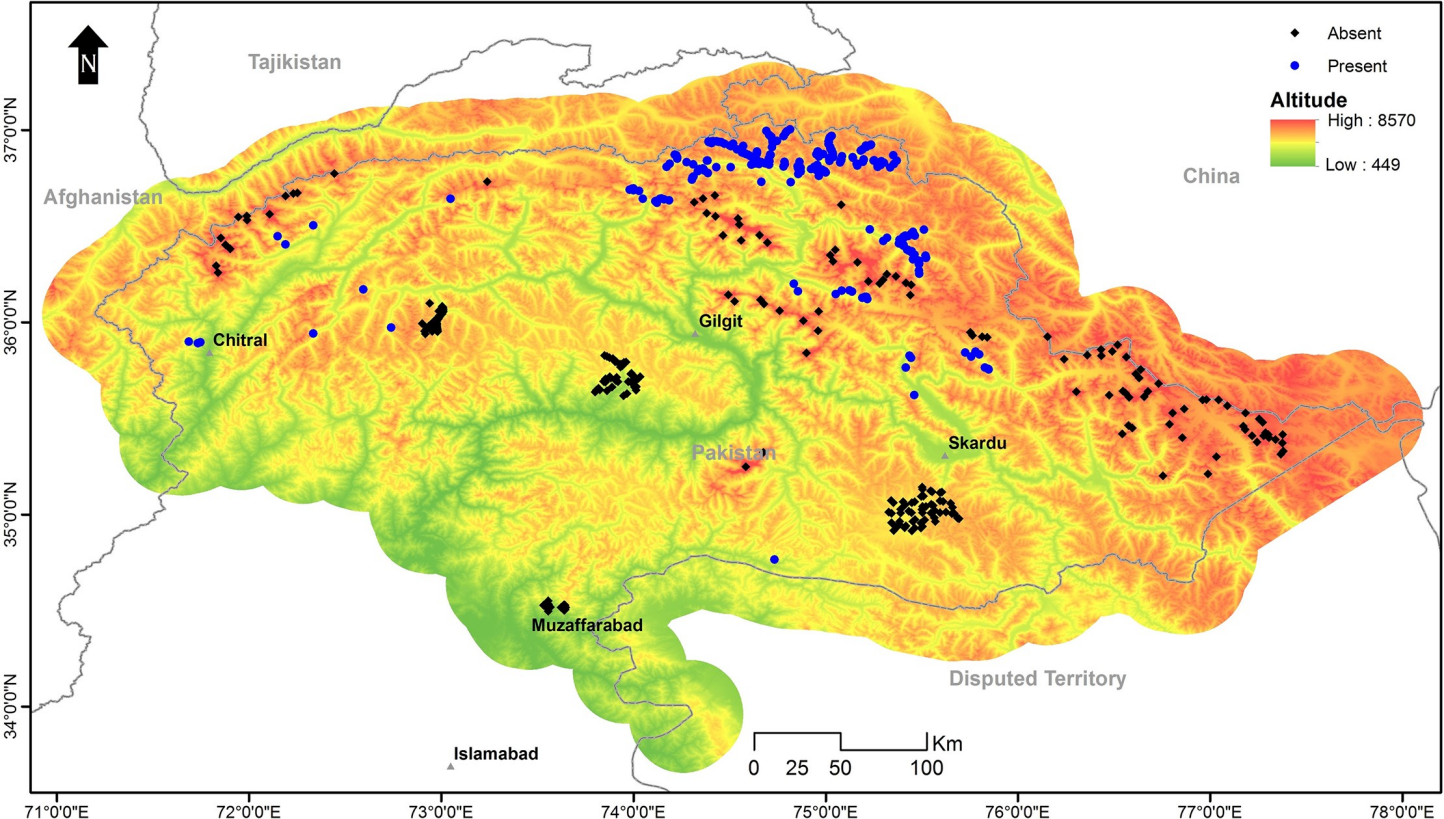
Presence and absence locations of snow leopards used for model evaluation.

Due to the scarcity of snow leopard presence data, it was not possible to get a new presence record for model validation. Therefore, presence locations excluded by the collinearity model were used for model evaluation along with absence locations. Absence locations were obtained in two ways, a) from surveyed sites where snow leopards were not detected (214 locations), and b) through 102 locations which were extracted from areas higher than 6,500 m—no-go areas for snow leopards. Although using these locations was not ideal but the only option, we had, to get almost confirmed absence location from the study area [8] (Fig 3).

### Modelling potential movement corridors

Using the snow leopard suitability map generated by MaxEnt, we also modelled for potential movement corridors. This was achieved through Circuitscape 4.0 (software) [65], an open-source program that uses circuit theory to predict connectivity in heterogeneous landscapes for individual movement. The landscape is treated as a conductance surface by Circuitscape, where each pixel represents a resistor with an assigned resistance value. Pairwise electrical resistances between locations are calculated by running a theoretical electrical current between each population pair, with one population being set as the current source and the other as the ground [65]. We used Circuitscape because it has not only the strength to describe both wildlife movement [66] and gene flow [67], but also due to its capacity to describe probabilities of habitat connectivity for both small and large-scale landscapes. Circuitscape is based on random walks and does not assume that animals disperse according to previous knowledge of the surroundings other least cost resistance methods do [65]. It thus links populations through multiple pathways [65], such that connectivity between habitat patches increases according to the number of connected pathways, and the effective resistance between two populations is derived from the overall resistance across all pathways [48]. Though Circuitscape is often unable to compute grids larger than 6 million cells because of computer memory limitations [68] but it was fine for our study area. Also, a feature of current density maps produced by Circuitscape is that relatively high current is produced near the nodes is unwanted when there is no priority to place nodes in a particular location [69] but this did not affect our output as we have already chosen node locations.

We used our habitat suitability output as a conductance layer and 38 nodes to run movement corridors of snow leopard. These nodes represent different areas where we have confirmed snow leopard presence in Pakistan. We limited the number of nodes to 38 points, which cover all important areas of snow leopard in Pakistan, and not too nemrous to impart unnecessary complexity in the analysis. The nodes were converted into a grid file in Arc GIS 10.2, and both the habitat suitability map (Maxent output) and the nodes file were converted into ASCII format to run in Circuitscape model. We used the option of conductance instead of resistance because, in our model, higher values indicate greater ease of movement and we were interested in generating cumulative current maps [70–72]. Pairwise modelling mode was used which iterates across all pairs in a focal node. We connected the eight neighbouring cells, instead of four, as an average cost [69].

## Results

Snow leopard detection was low as it was photo-captured in 97 capture events at just 60 stations (out of 806 stations) (Fig 2). In most of our study areas, there was either single capture—Laspur Valley, Qurumber National Park, Musk Deer National Park, Terich Valley—or no capture (Broghil National Park, Deosai National Park, Yarkhun Valley, etc.). Multiple captures occurred only in the Khunjerab National Park, Shimshal, and Misgar valleys, Hopper-Hisper, and buffer areas of Central Karakoram National Park; Basha-Arandu.

In sign-based site-occupancy surveys, signs older than ten days were also excluded to minimize the risk of misidentification. After this screening, we obtained 213 locations in different areas with fresh signs—either scrapes or pugmarks, or both. Among 1,000 faecal samples, a genetic analysis confirmed 111 to be of snow leopards. Combining all three methods, we obtained 384 (Fig 2) confirmed locations of snow leopards. These locations were overlapping in some areas where multiple surveys were conducted and after removing spatially correlated data points, 98 unrelated locations were used to generate the current SDM of the snow leopard.

### Range-wide habitat suitability

MaxEnt produced outputs for 25 replicates and averaged them into one model along with response curves and AUC. This average model was used for drawing inferences about habitat suitability and calculating potential movement corridors.

**Fig 4:**
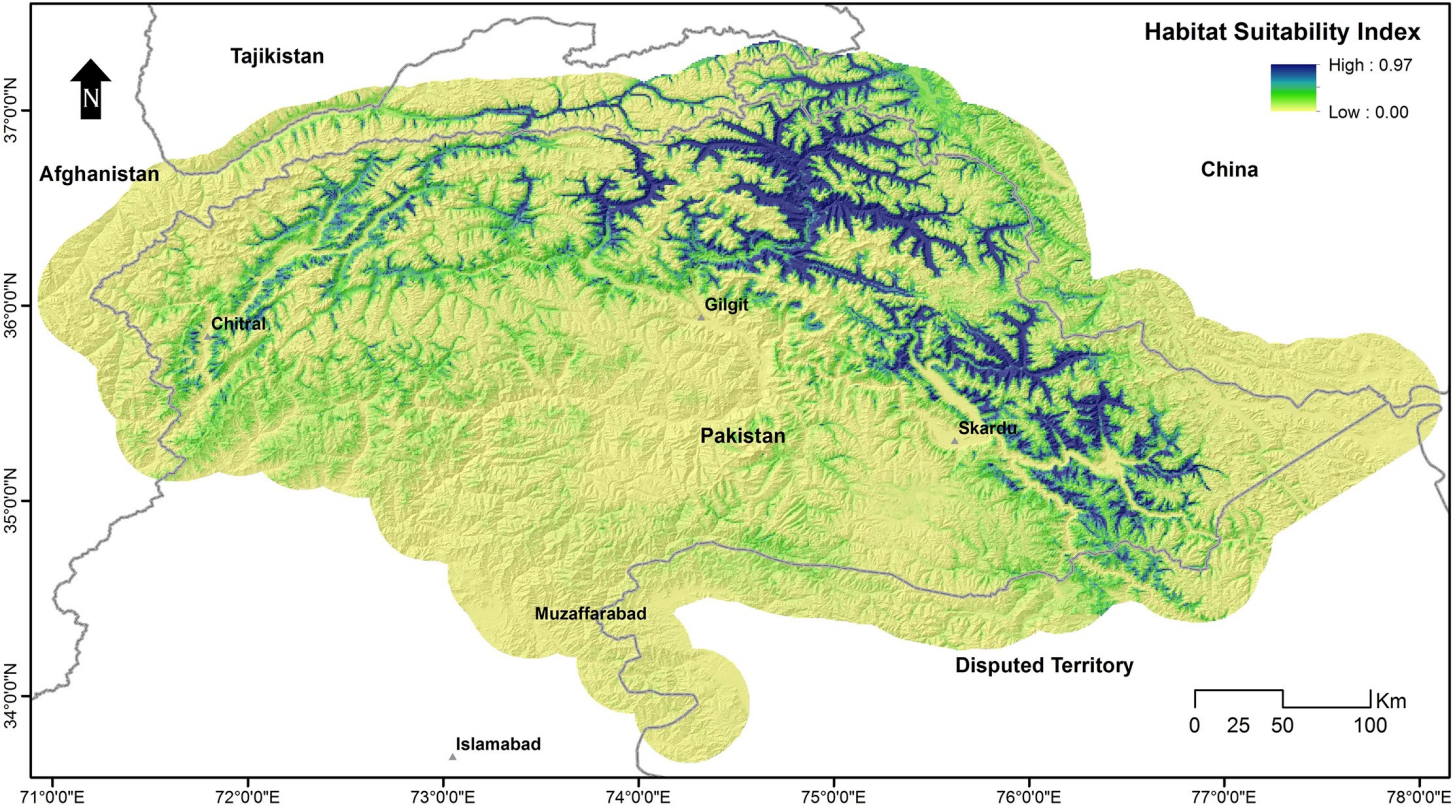
Habitat suitability of snow leopards in Pakistan, calculated with MaxEnt.

The habitat suitability score ranged from 0 to 0.97 across the snow leopard’s assumed range in Pakistan (Fig 4). A large portion of previously known range fell in low-quality habitat, including areas in lower Chitral, Swat, Astor, and AJK. Conversely, KNP, Misgar, Chapursan, Qurumber National Park, Broghil National Park, and CKNP contained high-quality habitat.

### Factors determining habitat suitability

Variables with higher contributions in the MaxEnt model were precipitation of driest month (34%), annual mean temperature (19.5%), mean diurnal range of temperature (9.8%), annual precipitation (9.4%), and river density (9.2). The contribution of other variables included in the model was low (Table 2).

**Table 2:** Estimates of relative contributions of the environmental variables to the Maxent model.

*Variable*	*Interpretation*	*Percent contribution*	*Permutation importance*
bio14	Precipitation of driest month	34	7.5
bio1	Annual mean temperature	19.5	21.8
bio2	Mean diurnal range (mean of monthly [max temp—min temp])	9.8	4.3
bio12	Annual precipitation	9.4	61.8
river	Density of rivers	9.2	0.2
road	Density of roads	5.6	2.5
soil	Soil	5.5	0.9
vrmint	Vector ruggedness measure	5.2	0.6
settlement	Density of Settlement	0.9	0.3
slope	Slope of the area	0.7	0.1
ndvi	Normalized difference vegetation index	0.2	0.1

**Fig 5:**
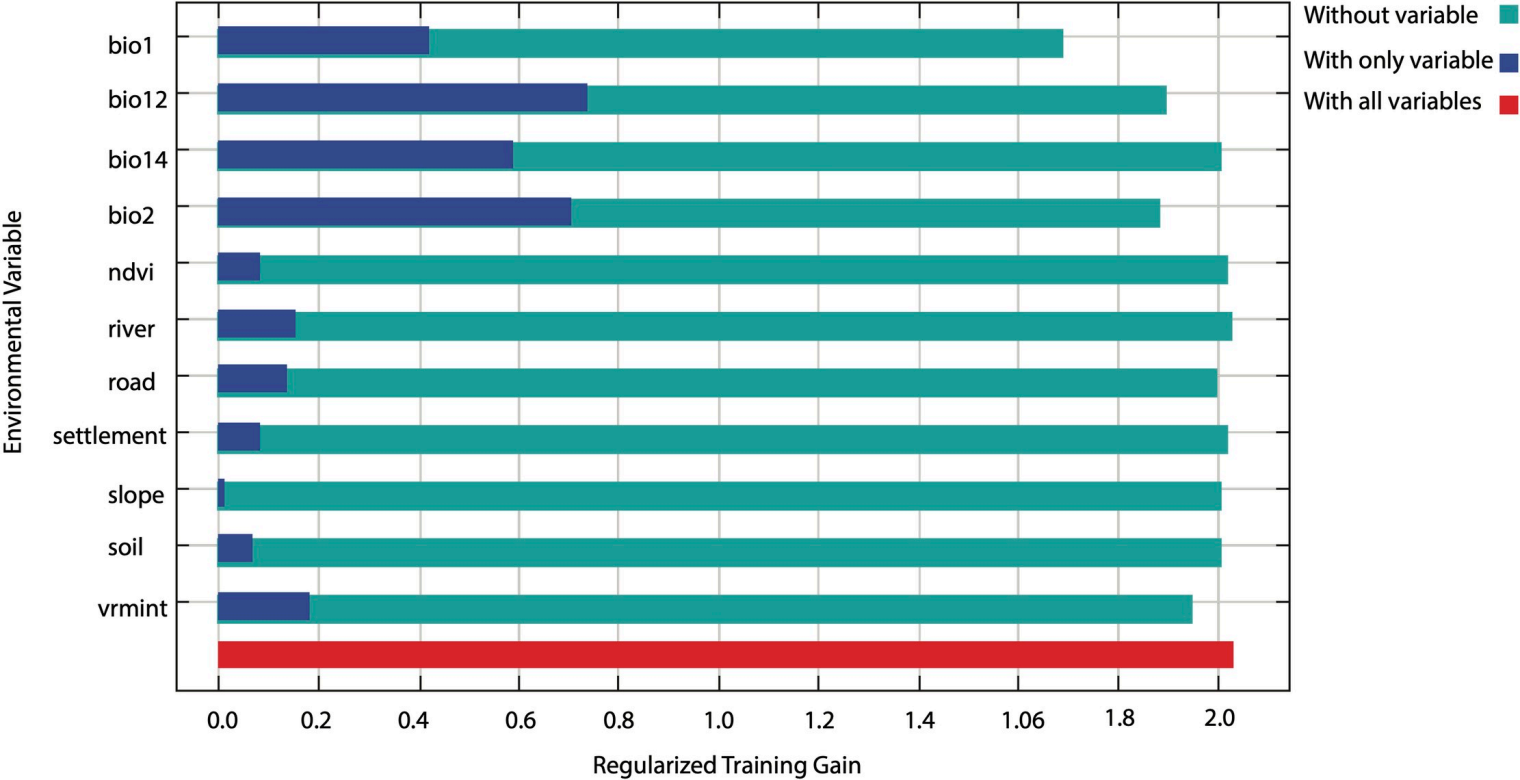
Jackknife test of regularized training gain of variables tested in snow leopard habitat suitability model. Blue bar = The gain when the environmental variable is used in isolation, Green bar = The gain when the environmental variable is omitted, Red bar = The gain with all environmental variables.

The Jackknife Test of variable importance showed that the environmental variable with the highest gain, when used in isolation, is the density of the river, which, therefore, appears to have the most useful information by itself. The environmental variable that decreased the gain the most when it was omitted was the annual mean temperature (bio1), which, therefore, appears to have the most information that is not present in other variables. The values shown are averages over replicate runs (Fig 5).

### Model evaluation and threshold selection

**Fig 6:**
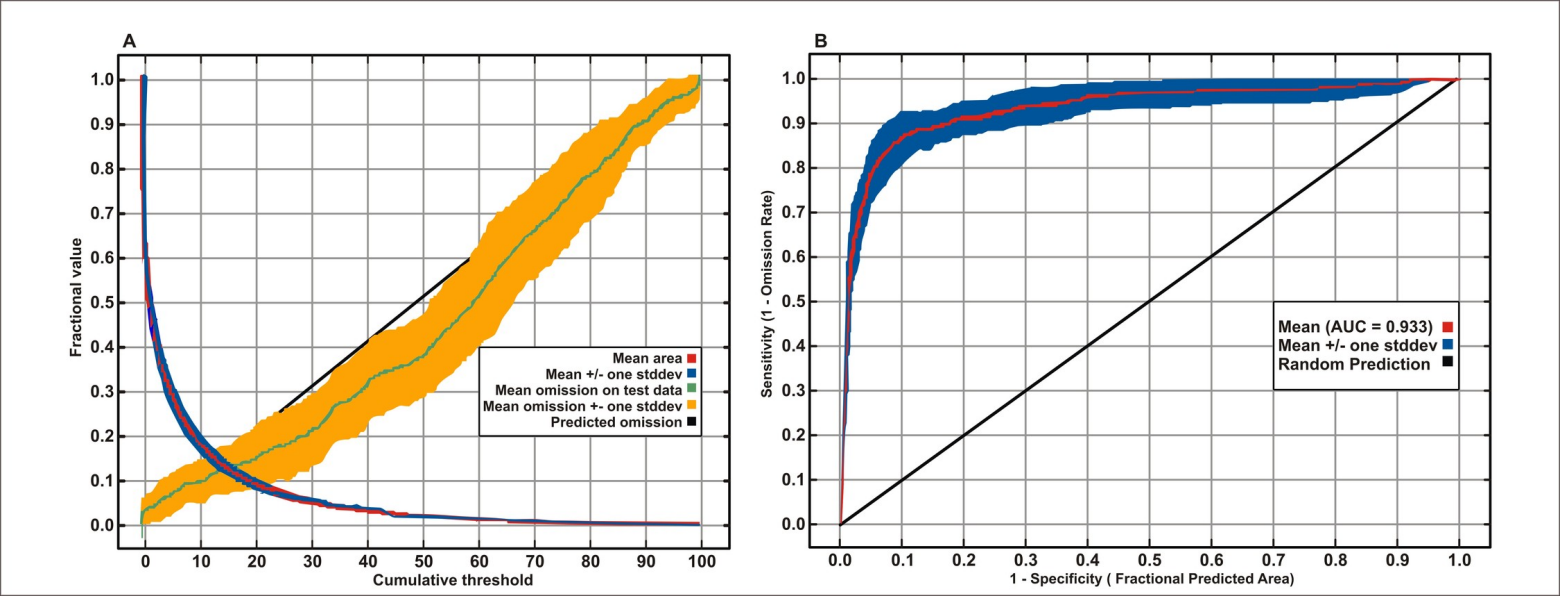
Model evaluations, (A) Averaged omission and predicted area for snow leopard, (B) The ROC curve calculated by MaxEnt as averaged sensitivity versus 1-specificity for snow leopard.

MaxEnt performed some basic statistics on the model and calculated an averaged AUC for the model. Analysis of omission/commission was done by MaxEnt and Fig 6A shows the test omission rate and predicted area as a function of the cumulative threshold averaged over the replicate runs. The omission rate should be close to the predicted omission because of the definition of the cumulative threshold and, in our case, is very close to the predicted one.

The ROC curve (Fig 6B) for the data was also calculated by MaxEnt, again, averaged over the replicate runs. Here, specificity is defined using the predicted area rather than true commission [30]. The average test AUC for the replicate runs was 0.933 and the standard deviation was 0.024.

**Fig 7:**
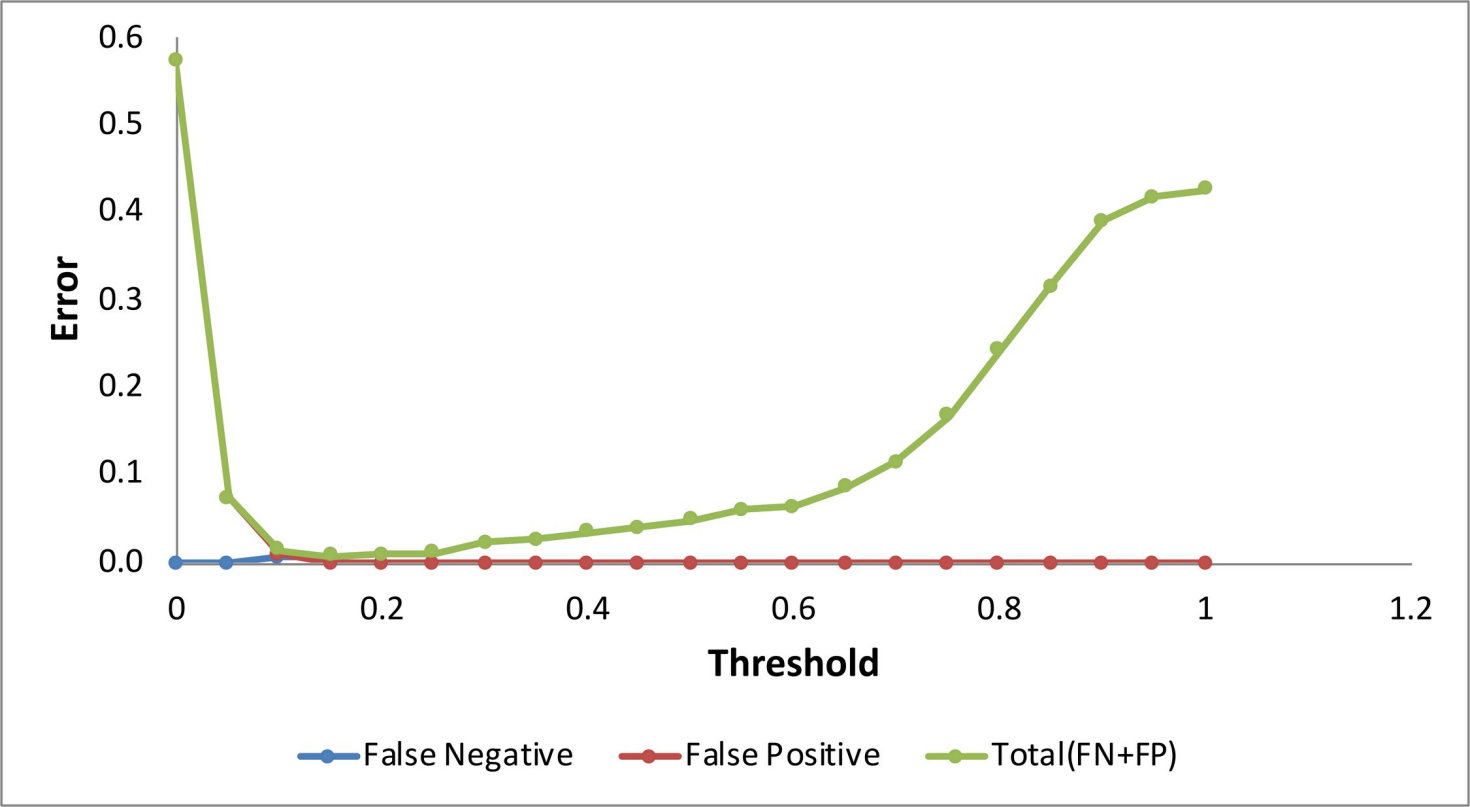
Graph showing the relationship of false negative and false positive rates against different thresholds of model prediction.

Measuring the error of false positive (FP) and false-negative (FN) rates against a range of defined thresholds (Fig 7), the lowest error was found at a threshold of 0.15. The binomial map was re-evaluated by plotting presence and absence points and it showed that almost all presence points were in suitable habitat areas and absence points in unsuitable areas. The values of 235 presence points and 316 absence points were extracted from the model and plotted against different thresholds. The value of AUC by ROC curve calculated at 0.15 was 1.000; which means our model performed very well.

It was calculated that 235 points were true positives (TPs) and 275 were true negatives (TNs), while FPs were 41 and FNs were 0. The true positive rate (TPR) was calculated at 1.000 while the false positive rate (FPR) was 0.130. Accuracy and specificity were calculated at 0.926 and 0.870, respectively, while the positive predictive value (PPV) was found to be 0.851 and the negative predictive value (NPV) was 1.000. The false discovery rate (FDR) was calculated at 0.149.

### Potential movement corridors of the snow leopard

The circuit model (Fig 9) revealed an interesting pattern concerning the snow leopard’s habitat connectivity. The population in the Hindukush landscape appears to be more connected with the population in Afghanistan as compared to other populations in Pakistan. Similarly, the Pamir-Karakoram population is better connected with China and Tajikistan, and the Himalayan population with the population in India.

We observed that Chitral had weak connections with other areas when we examined habitat connectivity in Pakistan. However, the populations of Phandar, Laspur Valley, and Yarkhun Valley seemed connected. Interestingly, Broghil National Park had a weak connection with its adjacent Qurumber National Park, but had strong links with Yarkhun Valley, while Qurumber National Park had strong links with Chapursan which is connected to Misgar, which had a strong link with KNP. The populations of CKNP and Musk Deer National Park were also shown to be isolated from others and the latter did not have any movement corridors close to it.

### Protected areas coverage in snow leopard’s habitat in Pakistan

**Fig 8:**
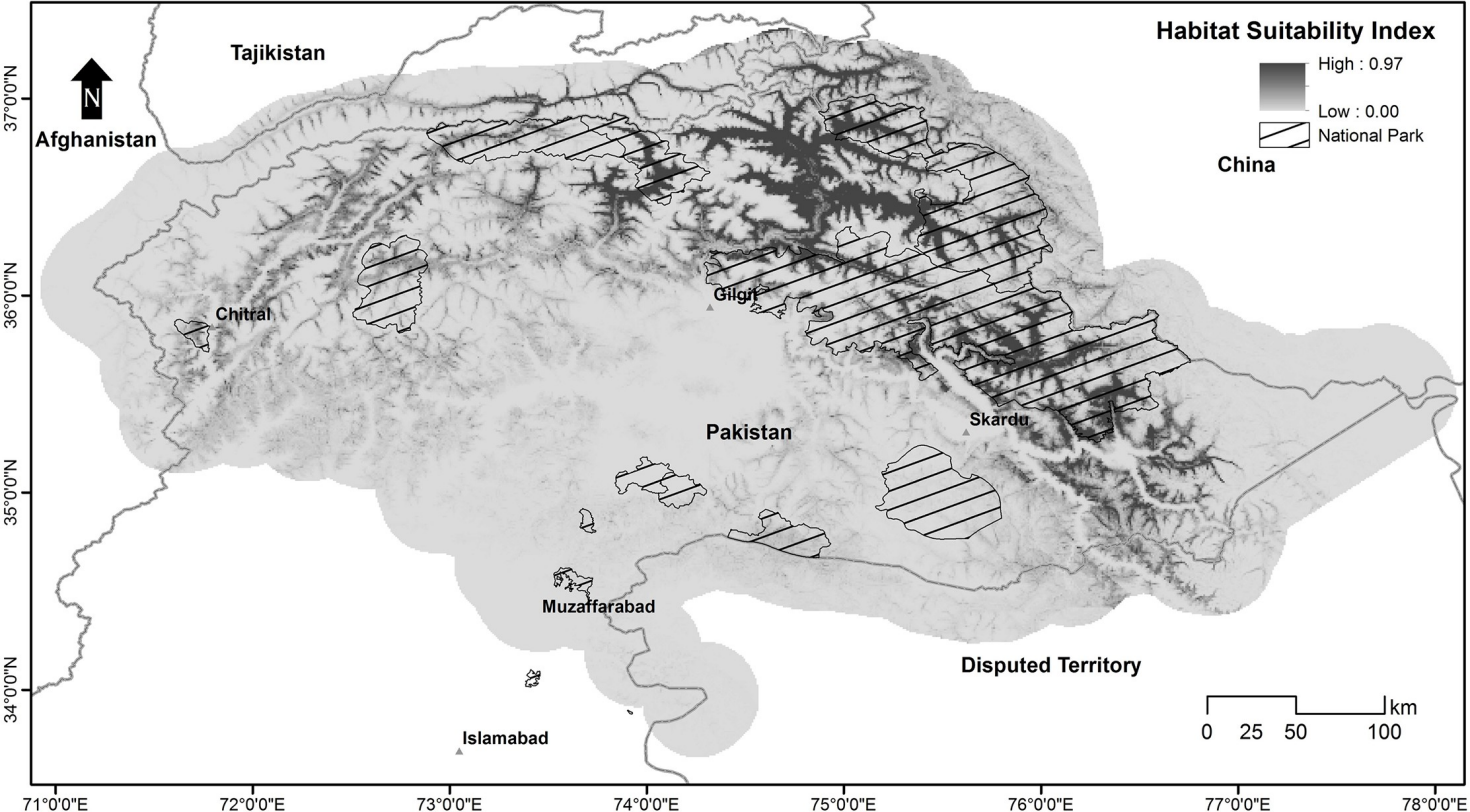
Overlay of existing national parks on habitat suitability map of snow leopards.

Habitat Suitability model was also assessed against current protected area coverage (Fig 8). Our analysis revealed that most of the suitable habitat of snow leopard in Pakistan has already been protected, however, there are some areas like Misgar, Chapursan, and Terich that are outside of any declared protected area.

**Fig 9:**
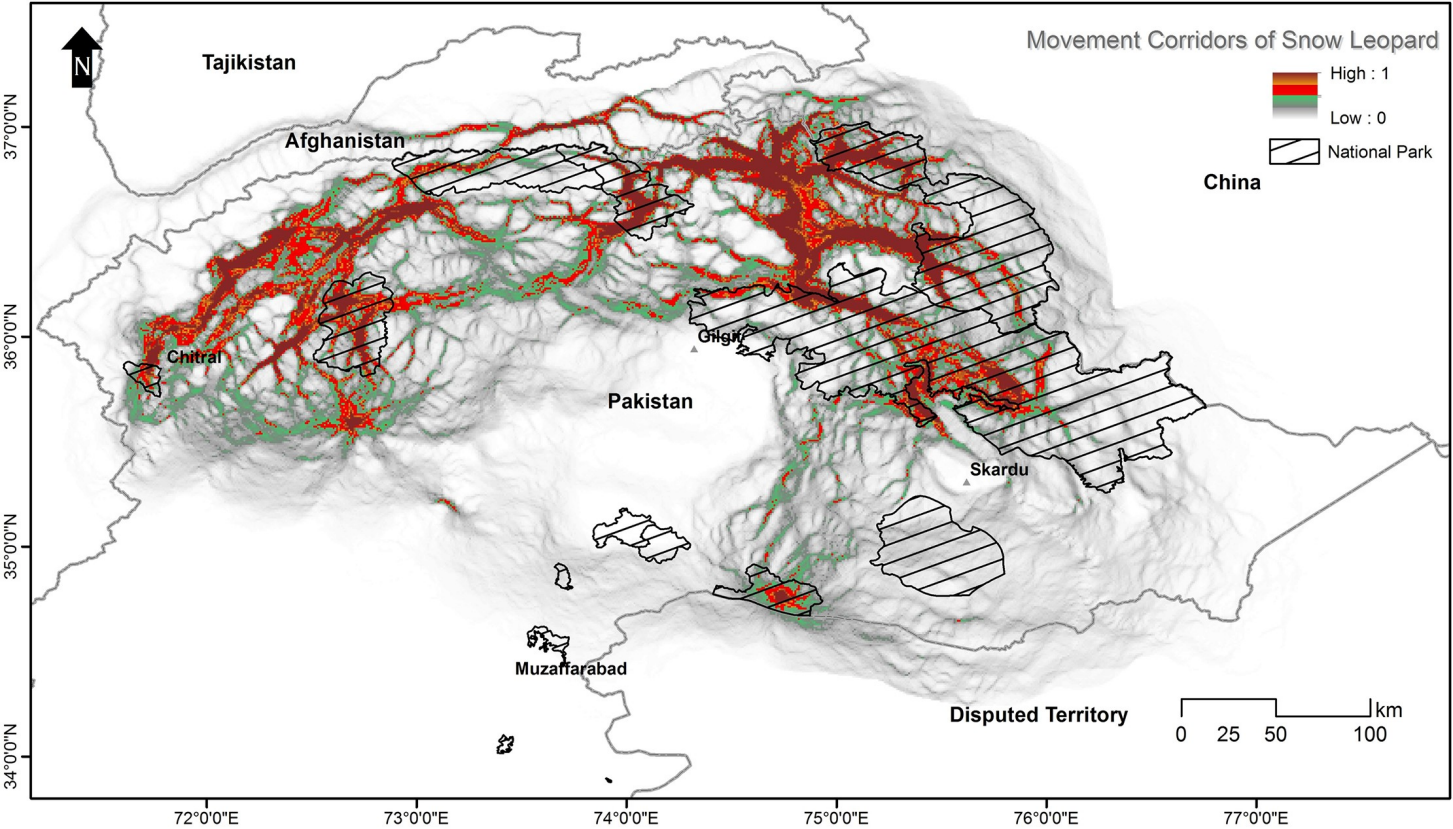
Potential movement corridors of snow leopards, calculated through circuitscape, between different national parks in northern Pakistan. Low values represent weak connectivity.

It was also observed that most of the national parks had weak links in regards to the movement of snow leopard across different habitats (Fig 9). Even some adjacent protected areas, like Broghil-Qurumber National Parks and Khujerab-Central Karakoram National Parks had no or very weak movement corridors of snow leopard at their shared borders.

## Discussion

Our study yielded the first empirically based inferences on snow leopard’s distributional patterns and habitat connectivity in Pakistan. We found that the distributional range estimated here does not correspond well with the ones described by Roberts [12] and Fox [13], which is not surprising given the elusive nature of the snow leopard and the lack of data available at the time. We recorded snow leopard presence using multiple techniques, including comparatively modern methods such as camera trapping and non-invasive genetic sampling which can be applied efficiently at large spatial scales. This allowed us to survey over 31,000km^2^ which covered about 39% of the presumed snow leopard range in Pakistan. The study showed on the one hand that the snow leopard range in Pakistan extends into areas beyond previously described distribution of the species [12, 13] and, on the other hand, that some areas previously believed to be part of the range either have very low suitability or are unsuitable.

This study showed that most of the snow leopard habitat in Pakistan is patchy, having no or weak links among the patches. Though there are potential movement corridors between different areas, e.g., between KNP and CKNP, but these are not strong enough to be called permanent routes (Fig 4). The connectivity model also revealed that in some areas, snow leopard possibly favoured movement across borders instead of inside Pakistan, e.g., Broghil National Park had more connectivity to Afghanistan than to its adjacent national park, Qurumber National Park. Also, KNP and CKNP did not show any connectivity at their shared border, but there is a movement corridor between these two parks via Hopper-Hisper valleys through Gojal area. These connectivity patterns seem unusual on maps, but other factors like the presence of large glaciers explain the absence of any movement corridors at the borders of these parks. The connectivity model proposed by McRae et al. [43] applying electrical circuit theory is a useful addition to the approaches available to ecologists and conservation planners. Circuit theory can be applied to predict the movement patterns and probabilities of successful dispersal or mortality of random walkers moving across complex landscapes, to generate measures of connectivity or isolation of habitat patches, populations, or protected areas, and to identify important connective elements (e.g., corridors) for conservation planning [43]. The establishment of movement corridors can offset the negative effects of habitat fragmentation by connecting isolated habitat populations or patches [73, 74]. Nevertheless, core habitats shall remain a priority for protection as they sustain viable populations. Corridors facilitate the movement of animals across larger landscapes, particularly through fragmented and less suitable areas, to maintain gene flow and connectivity among populations at the regional level.

Our habitat suitability model was also useful for assessing the effectiveness of existing protected areas, specifically national parks in the snow leopard’s habitat. Although a substantial proportion of suitable snow leopard habitat in Pakistan falls in national parks, there are still many areas that should be considered for inclusion in the protected area's network (Fig 8), to safeguard the future of the species. Misgar and Chapursan falling between KNP and Qurumber National Park are some of the most suitable areas for snow leopards still without protection. Areas on the eastern side of CKNP are also not protected. Qurumber National Park is unique in the sense that its entire area is favorable for snow leopards. But there should be a new protected area or extension of Qurumber National Park on its southern and southwest side. Yasin Valley is another important area adjacent to the southern side of Broghil National Park that requires protection. The upper part of the Chitral district in KP province is also suitable for snow leopards yet in need of protection.

There are two main limitations to our model. First, no estimates of prey population are available, which could have improved model predictions. Secondly, the low detection of snow leopard in the majority of the surveyed areas, resulted in scarce data, though this is typical for species like snow leopards.

### Management implications

**Fig 10:**
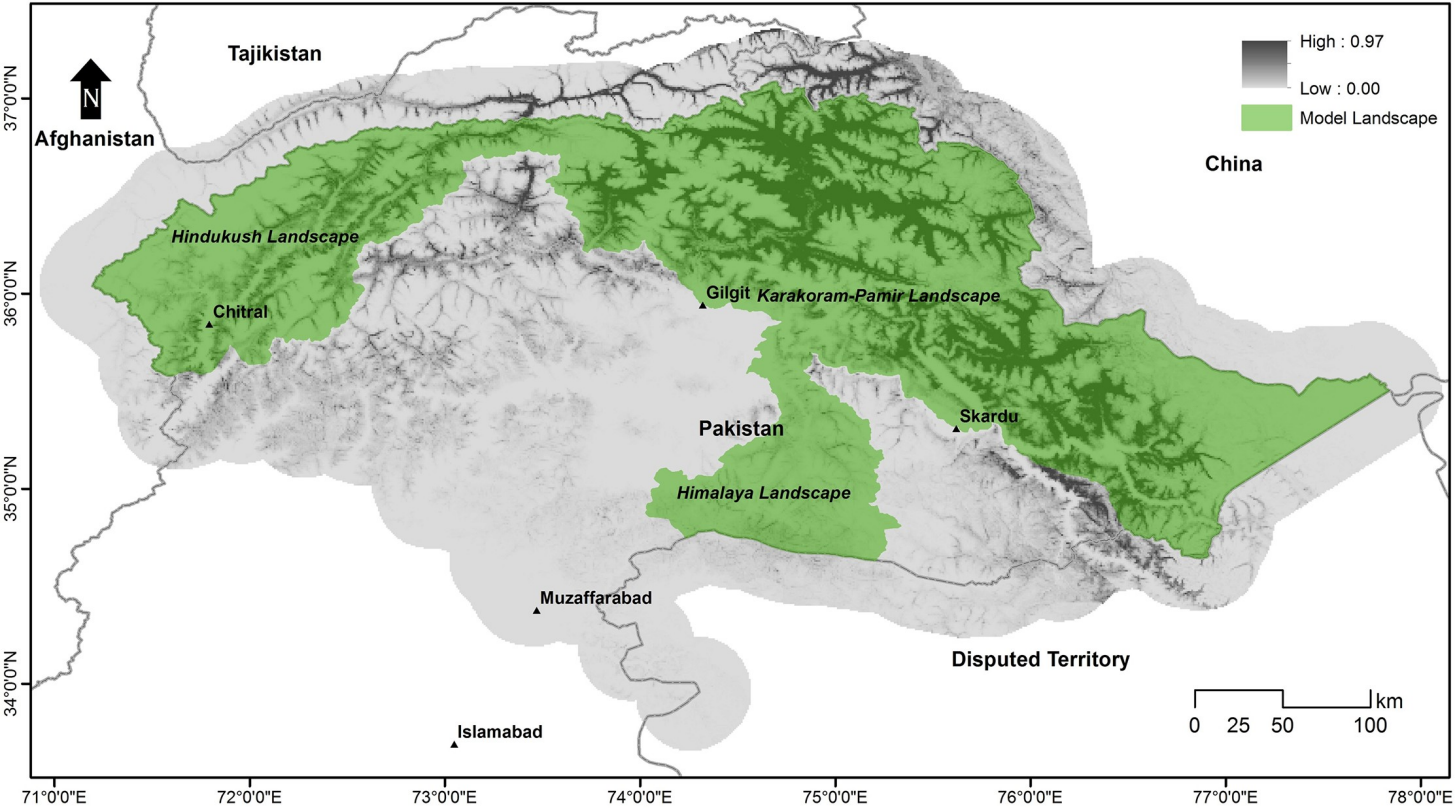
Recommended model landscapes for adoption under Global Snow Leopard and Ecosystem Protection Program (GSLEP).

The Global Snow Leopard Ecosystem Protection Program (GSLEP) is a joint initiative of 12 snow leopard range countries, established to safeguard snow leopards and the vast ecosystem. The overall aim of GSLEP is to secure at least 20 snow leopard landscapes (SLL) across the cat’s range [33]. Among these 20 model landscapes, three were proposed in Pakistan. Each SLL is defined as an area that can support at least 100 snow leopards of breeding age, has adequate and stable prey populations, and has functional connectivity to other snow leopard landscapes, including across international boundaries [33]. However, in reality, the definition of these landscapes is theoretical, and their boundaries are delineated using limited information except for a few areas where empirical data were available. Our study allows us to propose three model landscapes to be included in the GSLEP agenda, based on habitat suitability of the snow leopards across Pakistan. These are named after mountain ranges they fall in; Himalaya, Karakoram-Pamir, and Hindukush (Fig 10). We also recommend that the Government of Pakistan shall establish new national parks to protect critical habitats of snow leopards falling in Misgar, Chapursan, and Terichmir areas in Gilgit-Baltistan and Chitral.

The geographic extents (km^2^) of three proposed model landscapes are; Himalaya = 7055, Karakoram = 38,245, Hindukush = 13,883. The snow leopard densities reported in the past studies range between 0.14–8.7 (average = 2.0) individual per 100 km^2^ [75, 76]. Density estimated in one part of the Karakoram range is 0.55 animals/100 km^2^ [77]. Though density estimates for the proposed landscapes are not available, each landscape is expected to support a sizeable population of snow leopards in view of the aforementioned densities from the region. All three landscapes also host good populations of prey species. For example, abundant prey in the Karakoram landscape is Himalayan ibex, though smaller populations of Ladakh urial, markhor, and blue sheep are also available. Similarly, the Himalaya landscape has populations of ibex and musk deer. Hindu Kush landscape supports populations of ibex and markhor. These landscapes also provide connectivity of the snow leopards populations with regional populations. For instance, the Himalaya landscape provides connectivity with the cat population in India on eastern side and connects with the Karakoram in the north-west. The Karakoram landscape provides wider connectivity with the populations in China in north and connects to Hindukush in the west. The Hindukush provides connectivity with the Central Asian populations through Afghanistan and Tajikistan. These factors justify these areas to be model landscapes for snow leopards in Pakistan.
